# Intensive Cardiac Rehabilitation Outcomes in Patients with Heart Failure

**DOI:** 10.3390/jcm12216886

**Published:** 2023-10-31

**Authors:** S. Hammad Jafri, Maya Guglin, Roopa Rao, Onyedika Ilonze, Kareem Ballut, Zulfiqar Qutrio Baloch, Mohammed Qintar, Joel Cohn, Matthew Wilcox, Andrew M. Freeman, Dinesh K. Kalra, Wen-Chih Wu

**Affiliations:** 1 Division of Cardiology, University of Louisville, 201 Abraham Flexner Way, Louisville, KY 40202, USA; 2 Division of Cardiovascular Medicine, Krannert Cardiovascular Research Center, Indiana University, Indianapolis, IN 46202, USA; 3 Department of Medicine Providence, Veterans Affairs Medical Center, Providence VAMC, 830 Chalkstone Ave, Providence, RI 02908, USA; wen-chih.wu@va.gov; 4 Department of Medicine, Providence VAMC, Alpert Medical School, Brown University, Providence, RI 02908, USA; 5 Sparrow Health System, Lansing, MI 48912, USAmatthew.wilcox@sparrow.org (M.W.); 6 Division of Cardiology, Department of Medicine, National Jewish Health, Denver, CO 80206, USA

**Keywords:** heart failure, guideline-directed medical therapy, cardiac rehabilitation, intensive cardiac rehabilitation, fitness, weight loss

## Abstract

Introduction: Cardiac rehabilitation (CR) has proven to be beneficial for patients with heart failure (HF), potentially reducing morbidity and mortality while improving fitness and psychological outcomes. Intensive cardiac rehabilitation (ICR) represents an emerging form of CR that has demonstrated advantages for patients with various cardiovascular diseases. Nevertheless, the specific outcomes of ICR in patients with HF remain unknown. Objectives: The purpose of this study is to assess the effectiveness of ICR in patients with HF. Methods: This retrospective study involved 12,950 patients who participated in ICR at 46 centers from January 2016 to December 2020. Patients were categorized into two groups: the HF group, comprising 1400 patients (11%), and the non-HF group, consisting of 11,550 patients (89%). The primary endpoints included the ICR completion rate, changes in body mass index (BMI), exercise minutes per week (EMW), and depression scores (CESD). A *t*-test was employed to compare variables between the two groups. Results: The HF group comprises older patients, with 37% being females (compared to 44% in the non-HF group). The ICR completion rate was higher in the non-HF group. After ICR completion, adjusted analyses revealed that patients without HF demonstrated a greater improvement in BMI. There were no differences in fitness, as measured via EMW, or in depression scores, as measured via CESD, between the two groups. Conclusions: Despite the lower baseline functional status and psychosocial scores of HF patients compared to non-HF patients, patients with HF were able to attain similar or even better functional and psychosocial outcomes after ICR.

## 1. Introduction

Patients suffering from heart failure (HF) experience considerable morbidity and mortality [1]. Various strategies have been explored to alleviate both the morbidity and mortality associated with HF, but they remain a significant burden on patients and the healthcare system [2,3]. Clinical trials have indicated that both cardiac rehabilitation (CR) and guideline-directed medical therapy (GDMT) are essential for secondary cardiovascular disease (CVD) prevention, and they can play a pivotal role in reducing morbidity and mortality, as well as enhancing physical fitness and psychological well-being in HF [4,5,6,7]. Despite the increasing adoption of GDMT for HF patients in recent years [8,9], there remains an underutilization of exercise training CR [10], even though they are recommended as Class I and IIa interventions, respectively, in national guidelines [11].

Intensive cardiac rehabilitation (ICR) represents a more comprehensive and non-pharmacological approach to CR, placing significant emphasis on non-exercise components [12,13,14,15,16,17]. ICR focuses on lifestyle modifications, notably adopting a low-fat, plant-based diet, which can exert a favorable influence on secondary prevention of cardiovascular diseases. ICR includes dedicated stress management, peer support, and dietary modifications, all in addition to exercise. ICR has demonstrated its advantages for patients dealing with a wide range of CVD [12,18]. Nevertheless, the outcomes of ICR for patients with HF remain unknown.

There are three ICR programs (Ornish [19], Pritikin [20], and Benson [21]) available for eligible patients. The Dean Ornish Cardiac Rehabilitation Program is structured around a stringent, plant-based diet that is low in fat and emphasizes whole grains, legumes, fruits, vegetables, and non-fat dairy [19]. Its primary goal is to lower cholesterol levels and enhance heart health through dietary choices. The program includes a fitness regimen that encompasses both aerobic and strength training exercises to enhance cardiovascular fitness. Stress reduction is a key focus, involving practices such as meditation and relaxation techniques. Additionally, participants are often encouraged to engage in group support to facilitate the adoption and sustenance of lifestyle changes. The Pritikin Program [20], just like Dean Ornish’s approach, places a strong emphasis on a plant-based, low-fat diet, with a particular focus on whole, unprocessed foods and the reduction of added sugars. It advocates regular physical activity to enhance cardiovascular well-being and overall fitness. In addition, this program prioritizes education, empowering participants with the information needed to make informed decisions about their nutrition, fitness, and overall health. Additionally, it frequently includes strategies for managing weight effectively, enabling individuals to achieve and maintain a healthy weight. Benson’s program is renowned for its focus on mind–body approaches, notably the relaxation response, aimed at stress reduction and fostering relaxation [21]. It promotes the adoption of a healthier lifestyle, encompassing dietary improvements, regular physical activity, and effective stress management. Central to the program are practices like meditation and relaxation exercises, which play a pivotal role in stress management and overall well-being enhancement. Additionally, the program may adopt a more personalized approach, tailoring interventions to the unique requirements and preferences of each participant.

Although all three abovementioned ICR programs have received approval for payment from federal payers, their utilization remains relatively low in the broader population, particularly among HF patients.

Considering the limited availability of real-world outcomes data for ICR in HF patients, our objective is to examine and contrast the outcomes of ICR between HF patients and those without HF, using a nationwide sample of individuals participating in the Ornish ICR program [19].

## 2. Methods

### 2.1. Data Collection and Study Design

We carried out a retrospective cohort study that included a total of 12,950 patients who participated in the Dean Ornish ICR program from January 2016 to December 2020 from 46 locations across the United States. Data were collected from Ornish registry and maintained by Sharecare, Inc. Information regarding program completion, as well as psychosocial and fitness outcomes for those who successfully completed the program, was collected from the Ornish ICR registry. These data were anonymized and made available to the researchers for analysis. This study was granted an exemption due to its use of de-identified data by the Institutional Review Board (IRB) at the Providence VA Medical Center. Upon data collection, a comparison was made between the specific outcomes in patients with HF and those without HF both before and after their participation in CR.

### 2.2. Patient Selection

Patients were categorized as having HF if they were enrolled in the ICR program for the indication of HF or if their LVEF was less than 35%, as indicated in echocardiogram results. Patients without HF were those eligible for ICR due to following medical conditions: ST elevation myocardial infarction (STEMI), non-ST elevation myocardial infarction (NSTEMI), coronary artery bypass graft (CABG), percutaneous coronary intervention (PCI), angina, and valvular procedures.

### 2.3. Intensive Cardiac Rehabilitation and Exercise Prescription

ICR shares identical eligibility criteria with CR, encompassing patients who have undergone PCI, CABG, experienced NSTEMI/STEMI, suffer from heart failure with reduced ejection fraction, endure chronic angina, have undergone heart transplant, or have had valve repair/replacement, as stipulated in the American Heart Association (AHA)/AACVPR guidelines [11]. In the case of patients with heart failure, enrollment in ICR was contingent on a left ventricular ejection fraction (LVEF) of 35% or less.

The Dean Ornish ICR program consists of four one-hour sessions, held twice a week for a total of nine weeks. These ICR sessions are structured as follows: The first one-hour session is dedicated to supervised exercise, overseen by an exercise physiologist. Following that, there is a one-hour session for stress management activities such as meditation or yoga, led by a qualified instructor. Another one-hour session focuses on peer support, guided by a licensed therapist. Lastly, there is a one-hour session devoted to educational sessions about a plant-based diet led by a registered dietitian. These dietary sessions also include a sample meal provided by the ICR program. Notably, the ICR program features only one hour of exercise twice weekly for a total exercise time of 18 h over nine weeks, which is considerably shorter than the standard CR program with a 36 h duration dedicated entirely to exercise.

### 2.4. Outcome Measurements

The primary endpoints included the ICR completion rate, change in body mass index (BMI), exercise minutes per week (EMW), and depression scores as measured by the Center for Epidemiological Studies Depression (CESD). The ICR completion rate was defined as the patient’s completion of all prescribed sessions.

Body mass index (BMI) was calculated as weight in kilograms divided by the height in meter square. Height was collected at baseline, while weight was collected at baseline and during each ICR session.

Exercise minutes per week represents the total minutes of exercise recorded each week during the ICR sessions. We are using this measure as a surrogate for metabolic equivalents (METs) or a six-minute walking distance.

The Center for Epidemiological Studies-Depression (CESD) score is a well-validated, 20-item questionnaire commonly used in depression research. The score ranges from 0 to 60, where a higher score indicates more depression symptoms. A change of 7 points is considered clinically significant [22].

The secondary endpoints include changes in blood pressure (BP), cholesterol, low-density lipoprotein (LDL), triglycerides (TGL), and health status (SF-36 physical and mental composite scores).

SF-36 or Health-related quality of life: This is a well-validated and commonly used thirty-six-item questionnaire to measure physical and psychological well-being. It consists of eight domains: physical functioning, physical role limitations, pain, general health, vitality, social function, emotional role limitations, and mental health. These domains can be summarized into two composite scores: physical composite score (PCS) and mental composite score (MCS). The scoring ranges from 0 to 100, where a higher score indicates better health. A change of 5 points is considered clinically significant [23].

### 2.5. Covariate Assessment

A diagnosis of coronary artery disease (CAD), non-ST elevation myocardial infarction (NSTEMI) or ST elevation myocardial infraction (STEMI), percutaneous coronary intervention (PCI), coronary artery bypass grafting (CABG), heart failure (HF), angina, valve procedures, hypertension (HTN), diabetes mellitus (DM) and hyperlipidemia (HLP) was recorded via chart review upon entry into the ICR program and subsequently confirmed with the patient. Patients were categorized into low-, medium-, and high-risk categories per the American Association of Cardiovascular and Pulmonary Rehabilitation (AACVPR) risk category [11,24]. Prescribed medications were reviewed and confirmed with the patient at the start of the CR program. Smoking status was self-reported and included information on the number of cigarettes smoked, whether the patient had quit smoking or had never smoked in the past.

#### Statistical Analysis

Continuous variables were presented as mean ± standard deviation, while categorical variables were expressed as frequencies or percentages. Patients were stratified by the presence of heart failure (HF) for comparison, resulting in two groups: 1400 patients (11%) in the HF group and 11,550 patients (89%) in the non-HF group. A z-test was used to compare variables between these two groups. Linear regression was used to adjust for differences in baseline variables, including age, race, gender, BMI, blood pressure (BP), hypertension (HTN), hyperlipidemia (HLP), diabetes mellitus (DM), smoking, obesity, and AACVPR risk category. Logistic regression was utilized to construct a prediction model to identify variables independently associated with ICR completion. The multivariable adjustment model included age, gender, race, BMI, HTN, DM, and baseline values for SF-36, exercise minutes per week (EMW), and CESD scores. We conducted a retrospective power analysis to determine the required sample size for detecting a statistical difference between the two groups. With the sample size for the smaller arm already known, we calculated the sample size for the larger arm using the formula n_2_ = n_1_/1 − *p*. This resulted in a requirement of 1400 patients in each group when *p* is set to one to account for equal group sizes. We also utilized another formula to identify a significant 10% increase in the primary outcome between the two groups. This required a minimum of 385 patients in each group, with a statistical power of 80% and a significance level of 5%. It is worth mentioning that there was minimal missing data, impacting fewer than 5% of the patients. This missing data was primarily related to incomplete laboratory values that were not considered in the primary or secondary outcomes. A two-sided alpha level of ≤0.05 was considered statistically significant. Statistical analyses were conducted using the Stata statistical package (Stata 15.1).

## 3. Results

The mean age of the participants was 66.6 years, and 43% of them were females. The HF group consisted of older patients (HF: 68.5 ± 11 years vs. non-HF: 66.0 ± 11 years, *p* < 0.01), with 37% being females (compared to 44% females in the non-HF group) and 52% being of white ethnicity (compared to 50% whites in the non-HF group). Patients with HF were more likely to have a diagnosis of HTN, HLP, DM, and obesity compared to those without HF (Table 1). Patients with HF also have a lower baseline functional status, as measured using EMW and psychosocial scores (CESD and SF-36), at baseline compared to non-HF patients (Table 1).

The ICR completion rate was higher in the non-HF group compared to the HF group (HF: 63.8% vs. non-HF: 74.1%, *p* < 0.01). Following ICR completion, both patient groups demonstrated significant improvements in primary and secondary endpoints. Adjusted analyses revealed that patients without HF experienced a greater improvement in BMI (HF: −1.07 ± 1.81 vs. non-HF: −1.47 ± 1.58, *p* < 0.01) (Table 2). However, there was no difference in the change in fitness, as measured using EMW (HF: 99.84 ± 144.58 vs. non-HF: 100.17 ± 145.35, *p* = 0.95) or in depression scores measured using CESD between both groups (HF: −5.48 ± 8.12 vs. non-HF: −5.36 ± 8.36, *p* = 0.72) (Table 2).

The non-HF group exhibited a greater reduction in BP (HF: −3.68 ± 19.10 mmHg vs. non-HF: −5.30 ± 20.03 mmHg, *p* < 0.01), cholesterol levels (HF: −18.72 ± 38.43 mg /dL vs. non-HF: −24.66 ± 39.03 mg /dL, *p* = 0.04), and LDL (HF: −14.36 ± 32.90 mg /dL vs. non-HF: −18.96 ± 33.02 mg /dL, *p* < 0.01) compared to the HF group (Table 2). However, the HF group experienced greater improvements in SF-36 physical composite scores (HF: 6.75 ± 7.33 vs. non-HF: 5.44 ± 6.74, *p* < 0.01) than the non-HF group. There were no significant differences in TGL and SF36MCS between the two groups after completing ICR (Table 2).

Per a multivariable prediction model, advanced age, higher BMI, elevated EMW, and superior SF36PCS scores prior to initiating ICR were associated with an increased likelihood of completing ICR. Conversely, a diagnosis of HF and higher depression scores were linked to a reduced likelihood of completing ICR (Table 3).

## 4. Discussion

Our study has demonstrated that patients with HF experience similar improvements in fitness and depression levels after completing ICR in comparison to patients without HF. Despite the fact that completion rates for ICR were higher among non-HF patients than among those with HF, both groups achieved significant enhancements in CR outcomes post-ICR completion. Patients without HF exhibited greater improvements in BMI, BP, cholesterol, and LDL compared to the HF group. In contrast, patients in the HF group showed more substantial improvements in the physical component of their quality of life in comparison to the non-HF group. However, there were no significant differences in the changes in TGL and SF36MCS between the two groups following ICR completion.

The reasons behind the lower ICR completion rates for HFrEF in ICR are likely multifaceted. Factors such as lower overall functional status, psychosocial well-being, and a higher burden of comorbidities are probable contributors to this phenomenon. Consequently, our regression analysis unveiled that a diagnosis of HF and higher depression scores before ICR served as negative predictors for ICR completion. In contrast, older age, obesity, and higher physical fitness and physical quality of life scores (measured using SF36PCS) before ICR were linked to higher ICR completion rates. Although our study population consisted of older HF patients with lower ICR completion rates, they also displayed higher depression scores and lower quality of life scores (according to SF36PCS) before commencing cardiac rehabilitation. Consequently, these factors collectively contributed to the overall lower completion rates among the HF population. A previous study [18] has also indicated that older age is a significant predictor of ICR completion, thus confirming our study results. Conversely, a study of women in traditional CR showed that once enrolled in the program, HF patients were as likely as CAD patients to complete the program [25]. In light of these findings, future studies should prioritize exploring logistical strategies (such as offering a more flexible schedule, addressing transportation challenges, and potentially increasing staffing) to better support patients with HF, who often have lower functional status and a heavier burden of comorbidities, in attending and successfully completing ICR.

The progression of heart failure (HF) can culminate in end-stage HF, necessitating numerous hospitalizations, medication adjustments, and, ultimately, heart transplant or left ventricular assist device implantation. These interventions can place a substantial burden on both the healthcare system and the patient [26,27,28]. In addition to GDMT, clinical trials have demonstrated that CR can effectively reduce morbidity, mortality, and the advancement of the disease in HF [4,5,6,8]. Various strategies, including home-based CR, telemedicine, and other interventions, have been implemented with increasing success in improving CR completion rates [29,30,31]. We anticipate that ICR can serve as a potent adjunctive tool for HF patients, helping to slow the progression of the disease and enhance both physical and psychological well-being. Ultimately, this could lead to a reduction in morbidity and mortality.

Patients with HF frequently experience psychosocial limitations, such as depression and anxiety, which can significantly contribute to the non-completion of treatment [17]. Depression, in particular, can exacerbate HF due to several factors, including the patient’s reduced ability to engage in treatment programs or adhere to them [32]. Conversely, the diagnosis of HF can further worsen depression in affected individuals, making it a complex challenge to address both conditions simultaneously [33]. Given the heightened focus on addressing psychosocial components, ICR may offer greater potential for improving psychosocial symptoms compared to traditional CR, as demonstrated in our previous study [12]. Consequently, ICR represents an excellent opportunity for patients with HF to enhance their mental well-being and psychosocial functioning.

### Strengths and Limitations

Our study, conducted across multiple centers nationwide with a diverse and substantial sample size, represents one of the initial efforts to report on ICR outcomes in patients with HF. However, as an observational study, the possibility of residual confounding remains despite our rigorous attempts to account for baseline differences. Unfortunately, we did not have access to data on variables such as METs and maximal oxygen consumption, as well as rates of HF hospitalizations and mortality, which could have provided additional insights into the outcomes of ICR in HF patients. Additionally, it is important to note that individuals who chose to participate in ICR may have been more motivated, potentially limiting the generalizability of our findings beyond the specific ICR context.

## 5. Conclusions

Significant improvements in ICR outcomes were achieved for both groups, with and without HF. Despite the lower baseline functional status and psychosocial scores of HF patients compared to non-HF patients, patients with HF were able to achieve similar or even better functional and psychosocial outcomes after ICR. Future studies should investigate whether ICR offers unique advantages in psychosocial outcomes over traditional CR for patients with HF.

## Figures and Tables

**Table 1:** Clinical and demographic characteristics for patients with and without HF enrolled in intensive cardiac rehabilitation (N = 12,950).

	HF Patients (N = 1400)	Non-HF Patients (N = 11,550)	*p*-Value * for between-Group Comparisons
Age (years)	68.54 ± 10.71	66.04 ± 10.78	<0.01
Gender (Female)	515 (37%)	5080 (44%)	<0.01
Race/Ethnicity (White)	732 (52%)	5735 (50%)	<0.01
Body Mass Index (kg/m^2^)	32.32 ± 7.40	31.62 ± 7.15	<0.01
Systolic Blood Pressure (mmHg)	123.97 ± 19.50	127.82 ± 17.19	<0.01
Risk Category			<0.01
Low	174 (13%)	3928 (37%)	
Medium	488 (37%)	4742 (44%)	
High	661 (50%)	2060 (19%)	
Risk Factors			
Hypertension	1079 (77%)	8117 (70%)	<0.01
Diabetes Mellitus	580 (41%)	3388 (29%)	<0.01
Hyperlipidemia	8742 (76%)	1010 (72%)	<0.01
Obesity	732 (52%)	5688 (49%)	0.03
Current smoker	23 (2%)	119 (1%)	<0.04
Family hx heart disease	563 (40%)	4870 (42%)	0.16
Comorbid conditions			
PCI with and without stent	573 (41%)	4738 (41%)	0.54
STEMI/NSTEMI	3272 (28%)	564 (40%)	<0.01
CABG	331 (24%)	2056 (18%)	<0.01
Angina	250 (18%)	1934 (17%)	0.29
Heart Transplant	5 (0.4%)	13 (0.1%)	0.02
Valve repairs/replacements	144 (10%)	505 (4%)	<0.01
Completed cardiac rehabilitation	893 (63.8%)	8560 (74.1%)	<0.01
Number of sessions	57.90 ± 30.27	63.56 ± 27.98	<0.01
Total Cholesterol (mg /dL)	157.78 ± 55.03	166.78 ± 47.85	<0.01
Low-Density Lipoprotein (mg /dL)	85.13 ± 36.93	91.20 ± 39.76	<0.01
High-Density Lipoprotein (mg /dL)	45.59 ± 18.06	47.76 ± 14.86	<0.01
Triglycerides (mg /dL)	143.18 ± 107.37	147.93 ± 108.77	0.14
Exercise minutes per week	74.92 ± 119.33	98.70 ± 134.51	<0.01
CESD score	12.97 ± 10.59	11.76 ± 10.43	<0.01
SF36PCS	38.38 ± 9.90	45.28 ± 9.70	<0.01
SF36MCS	48.63 ± 10.16	49.21 ± 9.50	0.03

* *p*-value comparing two groups. Abbreviations: HF, heart failure; PCI, percutaneous coronary interventions; STEMI, ST elevation myocardial infarction; NSTEMI, non-ST elevation myocardial infarction; CABG, coronary artery bypass graft.

**Table 2:** Pre- and post-intensive cardiac rehabilitation values for patients with and without HF (N = 12,950).

	HF Patients (N = 1400) 11%	Non-HF Patients (N = 11,550) 89%	*p*-Value **
BMI (kg/m^2^)			
Pre-CR	32.32 ± 7.40	31.62 ± 7.15	<0.01
Post-CR	31.27 ± 7.03	30.08 ± 6.77	<0.01
Change	−1.07 ± 1.81 *	−1.47 ± 1.58 *	<0.01
SBP (mmHg)			
Pre-CR	123.97 ± 19.50	127.82 ± 17.19	<0.01
Post-CR	120.27 ± 17.03	122.50 ± 17.73	<0.01
Change	−3.68 ± 19.10 *	−5.30 ± 20.03 *	<0.01
Cholesterol (mg/dL)			
Pre-CR	157.78 ± 55.03	166.78 ± 47.85	<0.01
Post-CR	138.52 ± 37.86	143.05 ± 42.60	<0.01
Change	−18.72 ± 38.43 *	−24.66 ± 39.03 *	0.04
Low-Density Lipoprotein (mg/dL)			
Pre-CR	85.13 ± 36.93	91.20 ± 39.76	<0.01
Post-CR	70.78 ± 31.02	72.80 ± 33.76	<0.01
Change	−14.36 ± 32.90 *	−18.96 ± 33.02 *	<0.01
High-Density Lipoprotein (mg/dL)			
Pre-CR	45.59 ± 18.06	47.76 ± 14.86	<0.01
Post-CR	43.04 ± 13.13	45.17 ± 13.33	<0.01
Change	−2.65 ± 16.83 *	−2.88 ± 9.63 *	0.55
Triglycerides (mg/dL)			
Pre-CR	143.18 ± 107.37	147.93 ± 108.77	0.14
Post-CR	129.98 ± 74.96	130.47 ± 80.67	0.87
Change	−14.48 ± 94.29 *	−17.24 ± 83.77 *	0.39
Exercise minutes per week			
Pre-CR	74.92 ± 119.33	98.70 ± 134.51	<0.01
Post-CR	186.34 ± 140.75	202.69 ± 135.96	<0.01
Change	99.84 ± 144.58 *	100.17 ± 145.35 *	0.95
CESD score			
Pre-CR	12.97 ± 10.59	11.76 ± 10.43	<0.01
Post-CR	6.60 ± 7.55	5.75 ± 7.03	<0.01
Change	−5.48 ± 8.12 *	−5.36 ± 8.36 *	0.72
SF36PCS			
Pre-CR	38.38 ± 9.90	45.28 ± 9.70	<0.01
Post-CR	46.29 ± 9.19	51.55 ± 7.17	<0.01
Change	6.75 ± 7.33 *	5.44 ± 6.74 *	<0.01
SF36MCS			
Pre-CR	48.63 ± 10.16	49.21 ± 9.50	0.03
Post-CR	54.38 ± 7.31	54.79 ± 6.59	0.11
Change	4.80 ± 7.60 *	5.09 ± 7.76 *	0.33

* *p*-value < 0.01 among pre- and post-ICR; ** *p*-value comparing two groups; ** Adjusted *p*-values by regression modeling included the following variables: age, race, gender, BMI, hypertension, hyperlipidemia, diabetes mellitus, obesity, and AACVPR risk category; Abbreviations: HF, heart failure; CR, cardiac rehabilitation; CESD, center for epidemiologic studies depression; SF36MCS, short form-36 mental component summary; SF36PCS, short form-36 physical component summary.

**Table 3:** Prediction model using logistic regression for intensive cardiac rehabilitation completion (number of observations = 12,950).

Variables in Regression Model	Univariate Analysis (95% CI)	*p*-Value	Multivariable Analysis OR (95% CI)	Standard Error	Z	*p*-Value
Age	1.01 (1.00, 1.01)	<0.01	1.01 (1.01–1.02)	0.002	7.08	<0.01
Race	1.39 (1.30, 1.49)	<0.01	0.98 (0.89–1.07)	0.043	−0.44	0.65
Gender (male)	1.12 (1.05, 1.20)	<0.01	1.02 (0.93–1.11)	0.045	0.43	0.69
BMI	0.99 (0.98, 0.99)	<0.01	1.01 (1.01–1.02)	0.002	2.69	<0.01
Hypertension	1.32 (1.23, 1.41)	<0.01	0.96 (0.87–1.06)	0.048	−0.82	0.41
Diabetes Mellitus	0.98 (0.91, 1.05)	0.61	0.97 (0.88–1.07)	0.047	−0.63	0.53
Heart Failure	0.61 (0.55, 0.69)	<0.01	0.72 (0.63, 0.82)	0.048	−4.89	<0.01
Exercise minutes per week *	1.00 (1.00, 1.01)	<0.01	1.00 (1.00, 1.01)	0.001	2.37	0.02
CESD score *	0.98 (0.97, 0.98)	<0.01	0.99 (0.98, 0.99)	0.003	−3.24	0.01
SF36PCS *	1.02 (1.02, 1.03)	<0.01	1.03 (1.02, 1.03)	0.002	9.86	<0.01
SF36MCS *	1.13 (0.93, 1.37)	<0.01	0.99 (0.98, 1.01)	0.004	−1.49	0.14

Abbreviations: BMI, body mass index; CESD, center for epidemiologic studies depression; SF36MCS, short form-36 mental component summary; SF36PCS, short form-36 physical component summary; * Scores pre-intensive cardiac rehabilitation.

## Data Availability

Not applicable.

## Abbreviations

CR	Cardiac rehabilitation
ICR	Intensive cardiac rehabilitation
BMI	Body mass index
SBP	Systolic blood pressure
AACVPR	Association of Cardiovascular and Pulmonary Rehabilitation risk category
STEMI	ST elevation myocardial infraction
NSTEMI	Non-ST elevation myocardial infarction
PCI	Percutaneous coronary intervention
CABG	Coronary artery bypass graft
CHF	Congestive heart failure
CAD	Coronary artery disease
LVEF	Left ventricular ejection fraction

## References

[1] Long L., Mordi I.R., Bridges C., Sagar V.A., Davies E.J., Coats A.J., Dalal H., Rees K., Singh S.J., Taylor R.S. (2019). Exercise-based cardiac rehabilitation for adults with heart failure. Cochrane Database Syst. Rev..

[2] Benjamin E.J., Alonso A., Bittencourt M.S., Callaway C.W., Carson A.P., Chamberlain A.M., Chang A.R., Cheng S., Das S.R., Delling F.N. (2019). American Heart Association Council on Epidemiology and Prevention Statistics Committee and Stroke Statistics Subcommittee. Heart Disease and Stroke Statistics-2019 Update: A Report from the American Heart Association. Circulation.

[3] Braunwald E. (2015). The war against heart failure: The Lancet lecture. Lancet.

[4] Flynn K.E., Whellan D.J., Lin L., Blumenthal J.A., Ellis S.J., Fine L.J., Howlett J.G., Keteyian S.J., Kitzman D.W., Kraus W.E. (2009). Effects of exercise training on health status in patients with chronic heart failure: HF-ACTION randomized controlled trial. JAMA.

[5] Belardinelli R., Cianci G., Purcaro A. (1999). Randomized, controlled trial of long-term moderate exercise training in chronic heart failure: Effects on functional capacity, quality of life, and clinical outcome. Circulation.

[6] Taylor R.S., Walker S., Smart N.A., Piepoli M.F., Warren F.C., Ciani O., Whellan D., O’Connor C., Keteyian S.J., ExTraMATCH II Collaboration (2019). Impact of Exercise Rehabilitation on Exercise Capacity and Quality-of-Life in Heart Failure: Individual Participant Meta-Analysis. JACC.

[7] Ma W. (2004). AACVPR Guidelines for Cardiac Rehabilitation Programs & Secondary Prevention Programs.

[8] Blood A.J., Fischer C.M., Fera L.E., MacLean T.E., Smith K.V., Dunning J.R., Desai A.S., Bosque-Hamilton J.W., Aronson S.J., Gaziano T.A. (2020). Rationale and design of a navigator-driven remote optimization of guideline-directed medical therapy in patients with heart failure with reduced ejection fraction. Clin. Cardiol..

[9] Jafri S.H., Hushcha P., Dorbala P., Bousquet G., Lutfy C., Mellett L., Sonis L., Blankstein R., Cannon C., Plutzky J. (2023). Use of Optimal Medical Therapy in Patients With Cardiovascular Disease Undergoing Cardiac Rehabilitation. Curr. Probl. Cardiol..

[10] Alpert J.S. (2020). Cardiac Rehabilitation: An Underutilized Class I Treatment for Cardiovascular Disease. Am. J. Med..

[11] Thomas R.J., King M., Lui K., Oldridge N., Piña I.L., Spertus J. (2010). ACCFAHA Task Force on Performance Measures. AACVPR/ACCF/AHA 2010 Update: Performance measures on cardiac rehabilitation for referral to cardiac rehabilitation/secondary prevention services: A report of the American Association of Cardiovascular and Pulmonary Rehabilitation and the American College of Cardiology Foundation/American Heart Association Task Force on Performance Measures (Writing Committee to Develop Clinical Performance Measures for Cardiac Rehabilitation). J. Cardiopulm. Rehabil. Prev..

[12] Jafri S.H., Duazo C., Imran H., Bencie N.N., Imran T.F., Ahmad K., Deangelis J., Wu W.C. (2023). Physical and Psychological Outcomes of Patients Undergoing Traditional Cardiac Rehabilitation and Intensive Cardiac Rehabilitation. J. Cardiopulm. Rehabil. Prev..

[13] Detry J.M.R., Vierendeel I.A., Vanbutsele R.J., Robert A.R. (2001). Early short-term intensive cardiac rehabilitation induces positive results as long as one year after the acute coronary event: A prospective one-year controlled study. J. Cardiovasc. Risk.

[14] Katzenberg C., Silva E., Young M.J., Gilles G. (2018). Outcomes in a Community-Based Intensive Cardiac Rehabilitation Program: Comparison with Hospital-Based and Academic Programs. Am. J. Med..

[15] Malfatto G., Revera M., Branzi G., Ciambellotti F., Giglio A., Blengino S., Oldani M., Facchini C., Parati G., Facchini M. (2017). A brief period of intensive cardiac rehabilitation improves global longitudinal strain and diastolic function after a first uncomplicated myocardial infarction. Acta Cardiol..

[16] Świątkiewicz I., Di Somma S., De Fazio L., Mazzilli V., Taub P.R. (2021). Effectiveness of Intensive Cardiac Rehabilitation in High-Risk Patients with Cardiovascular Disease in Real-World Practice. Nutrients.

[17] Caccamo F., Saltini S., Carella E., Carlon R., Marogna C., Sava V. (2018). The measure of effectiveness of a short-term 2-week intensive Cardiac Rehabilitation program in decreasing levels of anxiety and depression. Monaldi Arch. Chest Dis..

[18] Jafri S.H., Ngamdu K.S., Price D., Baloch Z.Q., Cohn J., Wilcox M., Freeman A.M., Ornish D., Wu W.C. (2023). Intensive Cardiac Rehabilitation Attenuates the Gender Gap in Cardiac Rehabilitation Participation. Curr. Probl. Cardiol..

[19] Aldana S.G., Whitmer W.R., Greenlaw R., Avins A.L., Salberg A., Barnhurst M., Fellingham G., Lipsenthal L. (2003). Cardiovascular risk reductions associated with aggressive lifestyle modification and cardiac rehabilitation. Heart Lung.

[20] Máttar J.A., Salas C.E., Bernstein D.P., Lehr D., Bauer R. (1990). Hemodynamic changes after an intensive short-term exercise and nutrition program in hypertensive and obese patients with and without coronary artery disease. Arq. Bras. Cardiol..

[21] Razavi M., Fournier S., Shepard D.S., Ritter G., Strickler G.K., Stason W.B. (2014). Effects of lifestyle modification programs on cardiac risk factors. PLoS ONE.

[22] Van Dam N.T., Earleywine M. (2011). Validation of the Center for Epidemiologic Studies Depression Scale--Revised (CESD-R): Pragmatic depression assessment in the general population. Psychiatry Res..

[23] McHorney C.A., Ware Johne J.R., Raczek A.E. (1993). The MOS 36-Item Short-Form Health Survey (SF-36): II. Psychometric and clinical tests of validity in measuring physical and mental health constructs. Med. Care.

[24] Balady G.J., Ades P.A., Bittner V.A., Franklin B.A., Gordon N.F., Thomas R.J., Tomaselli G.F., Yancy C.W., American Heart Association Science Advisory and Coordinating Committee (2011). Referral, enrollment, and delivery of cardiac rehabilitation/secondary prevention programs at clinical centers and beyond: A presidential advisory from the American Heart Association. Circulation.

[25] Baig M., Imran H.M., Gaw A., Stabile L., Wu W.C. (2021). Cardiac rehabilitation in women; comparison of enrollment, adherence and outcomes between heart failure and coronary artery disease. Heart Lung.

[26] Shah K.S., Xu H., Matsouaka R.A., Bhatt D.L., Heidenreich P.A., Hernandez A.F., Fonarow G.C. (2017). Heart Failure With Preserved, Borderline, and Reduced Ejection Fraction: 5-Year Outcomes. J. Am. Coll. Cardiol..

[27] Guglin M., Zucker M.J., Borlaug B.A., Breen E., Cleveland J., Johnson M.R., Bozkurt B. (2020). ACC Heart Failure and Transplant Member Section and Leadership Council. Evaluation for Heart Transplantation and LVAD Implantation: JACC Council Perspectives. J. Am. Coll. Cardiol..

[28] Cain M.T., Firstenberg M.S., Cleveland J.C. (2021). Heart Transplant and Ventricular Assist: Cardiac Surgery and Heart Failure Perspective. US Cardiol. Rev..

[29] Aronov D., Bubnova M., Iosseliani D., Orekhov A. (2019). Clinical Efficacy of a Medical Centre- and Home-based Cardiac Rehabilitation Program for Patients with Coronary Heart Disease After Coronary Bypass Graft Surgery. Arch. Med. Res..

[30] Berry R., Brawner C.A., Kipa S.G., Stevens C., Bloom C., Keteyian S.J. (2020). Telemedicine Home-Based Cardiac Rehabilitation. J. Cardiopulm. Rehabil. Prev..

[31] Thomas R.J., Beatty A.L., Beckie T.M., Brewer L.C., Brown T.M., Forman D.E., Whooley M.A. (2019). Home-Based Cardiac Rehabilitation: A Scientific Statement From the American Association of Cardiovascular and Pulmonary Rehabilitation, the American Heart Association, and the American College of Cardiology. Circulation.

[32] Freedland K.E., Carney R.M., Rich M.W., Steinmeyer B.C., Rubin E.H. (2015). Cognitive Behavior Therapy for Depression and Self-Care in Heart Failure Patients: A Randomized Clinical Trial. JAMA Intern. Med..

[33] Chiala O., Vellone E., Klompstra L., Ortali G.A., Stroemberg A., Jaarsma T. (2018). Relationships between exercise capacity and anxiety, depression, and cognition in patients with heart failure. Heart Lung.

